# Association between relative handgrip strength and prediabetes among South Korean adults

**DOI:** 10.1371/journal.pone.0240027

**Published:** 2020-10-01

**Authors:** Bich Na Jang, Fatima Nari, Selin Kim, Eun-Cheol Park

**Affiliations:** 1 Department of Public Health, Graduate School, Yonsei University, Seoul, Republic of Korea; 2 Institute of Health Services Research, Yonsei University, Seoul, Republic of Korea; 3 Department of Preventive Medicine, Yonsei University College of Medicine, Seoul, Republic of Korea; San Raffaele Roma Open University, ITALY

## Abstract

**Background:**

Diabetes is a progressive disease, and thus, it is important to prevent diabetes at the prediabetes stage. Although the loss of muscle strength and prediabetes are associated, few studies have examined relative handgrip strength (RHGS), which can be an indicator of both muscle strength and adiposity. Therefore, our study aimed to examine the association between RHGS and prediabetes (HbA1c level >5.7%) stratified by sex due to sex differences in strength.

**Methods:**

We analyzed data from the 2016–2018 Korean National Health and Nutrition Examination Survey. Prediabetes was defined using the HbA1c cut-off level of 5.7–6.4%, identified by the American Diabetes Association. RHGS was calculated as the maximal absolute handgrip strength of both hands divided by body mass index and was divided into sex-specific quartiles. Multiple logistic regression analysis was performed to determine the association between sex-specific RHGS and prediabetes.

**Results:**

Among the total participants, 13,384 did not have diabetes. In men, the low and mid-low RHGS groups had increased odds of prediabetes (low group, odds ratio [OR]: 1.42, 95% confidence interval [CI]: 1.10–1.82; mid-low group, OR: 1.32, 95% CI: 1.04–1.67). However, no significant differences were observed between the corresponding female groups. Moreover, central obesity and lower RHGS were strongly associated with prediabetes in men (low group, OR: 2.40, 95% CI: 1.52–3.80; mid-low group, OR: 2.00, 95% CI: 1.26–3.17; mid-high group, OR: 1.76, 95% CI: 1.11–2.81), and a trend was observed (p = 0.0026).

**Conclusion:**

RHGS could be a practical and inexpensive tool for predicting diabetes in men. Programs aimed at preventing diabetes need to include exercise routines for improving muscle strength, and further research through longitudinal studies is required to investigate the causality of RHGS on the risk of prediabetes.

## Introduction

Diabetes is a non-communicable, progressive disease characterized by high blood glucose and elevated glycated hemoglobin (HbA1c) levels. According to the World Health Organization, 422 million people worldwide had diabetes in 2014 [1]. Compared to 1980, the age-specific prevalence of diabetes had doubled in men and was 60% higher in women in 2014 [2].

It is well known that obesity is a major risk factor for type 2 diabetes. One cohort study found that an increased body mass index (BMI) is associated with an increased risk of type 2 diabetes and that preventing obesity is linked to preventing diabetes [3]. Physical activity, which leads to energy expenditure, and skeletal muscle contraction can activate glucose metabolism and prevent obesity and diabetes [4] as well as strengthen muscles [5].

Some studies have shown that loss of muscle strength is associated with diabetes and that insulin resistance causes muscle protein loss [6, 7]. This is because skeletal muscle is the major site of insulin-mediated glucose uptake [8]. When muscle capacity decreases, insulin resistance increases, which can lead to diabetes progression. Previous studies found that patients with diabetes had low muscle strength [7, 9] and strengthening muscles via exercise has been reported to improve glycemic control in patients with diabetes [4, 10].

Handgrip strength is recommended as a simple and economical method for measuring muscle strength [11, 12], and absolute handgrip strength is the sum of the maximal measurement values of both hands [13]. Moreover, relative handgrip strength (RHGS), which considers BMI, can reflect both muscle strength and adiposity [11]. Thus, it is used as a more objective index than absolute handgrip strength.

It has been revealed that handgrip strength is a predictor of body protein loss and is linked to insulin resistance [14].One study of South Korean adults found an association between RHGS and diabetes diagnosed using fasting glucose levels [15]. Large population-based studies were also performed and found significant associations between RHGS and prediabetes [16, 17]. However, only a few studies have investigated the association between RHGS and prediabetes after adjusting for obesity.

We hypothesized that those with low RHGS were more likely to have an HbA1c level over 5.7% after adjusting for several health characteristics including obesity. Therefore, our study aimed to examine the association between RHGS and HbA1c levels, which reflect blood glucose levels [1], over a period of a few weeks. Moreover, due to sex differences in strengths [18], we investigated the differences in the prevalence of prediabetes according to RHGS between men and women.

## Methods

### Study population

This study used data from the Korea National Health and Nutrition Examination Survey (KNHANES). The KNHANES is a nationwide, population-based, cross-sectional survey assessing the health and nutritional status of the Korean population. It uses a stratified multistage cluster-sampling design to obtain a nationally representative sample. This survey has been conducted annually by the Korean Centers for Disease Control and Prevention since 1998. The data were not reviewed by an institutional review board on the basis of the Bioethics Act and Article 2 of its enforcement regulations. Furthermore, written consent was obtained from all participants before this survey. We conducted this study using the data from KNHANES Ⅶ (2016–2018).

**Fig 1:**
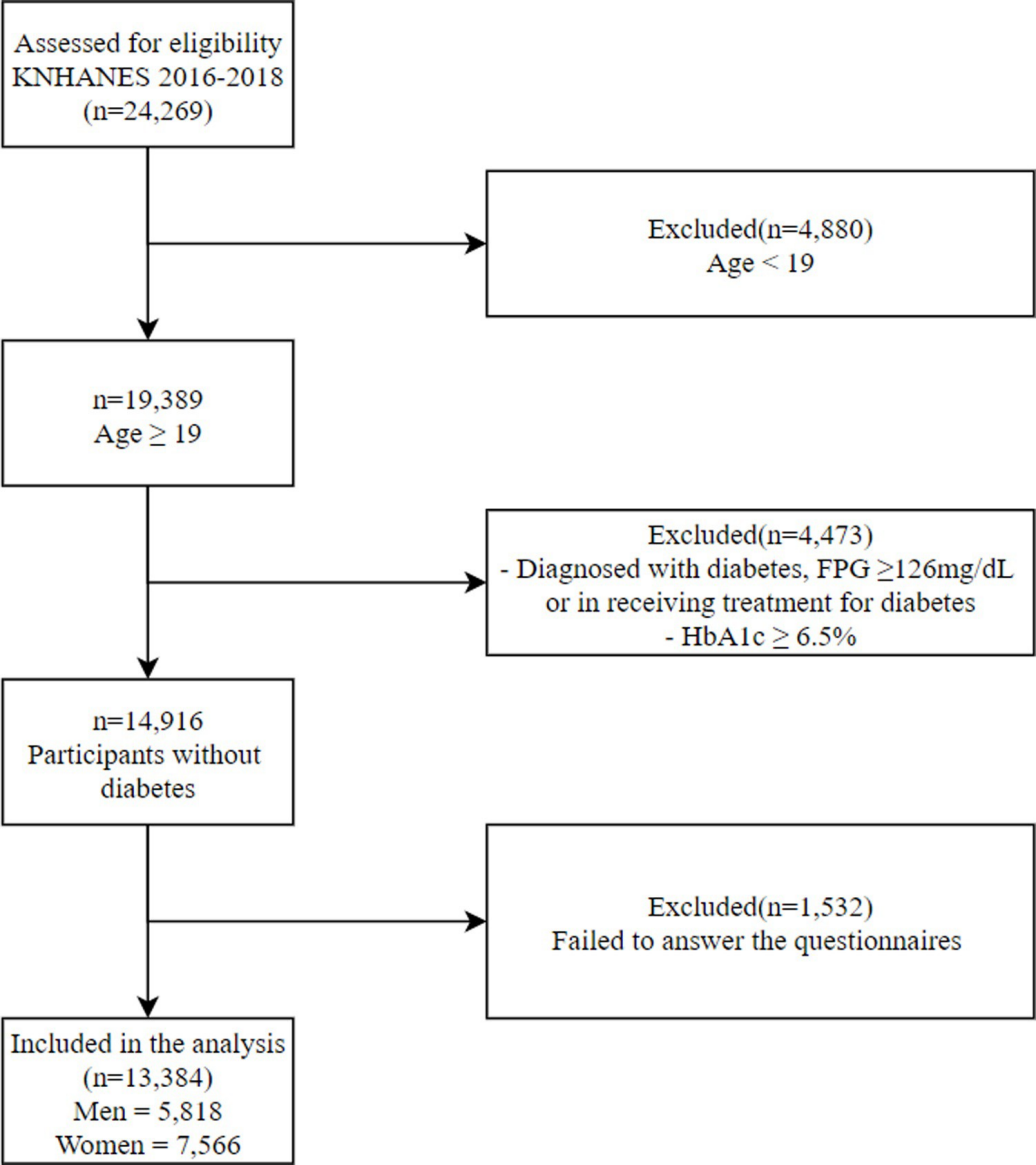
Flow diagram of subject inclusion and exclusion criteria. Abbreviations: KNHANES, Korea National Health and Nutrition Examination Survey; FPG, fasting plasma glucose.

We excluded participants aged under 19 years (n = 4,880) and those who were diagnosed with diabetes mellitus (fasting plasma glucose levels over 126 mg/dL, HbA1c levels over 6.5%, or the use of medications for diabetes mellitus) (n = 4,473) among the total subjects (n = 24,269). Furthermore, we excluded those without data for the variables included in our study (n = 1,532). Finally, 13,384 survey participants (5,818 men, 7,566 women) were selected for this study (Fig 1).

### Variables

The presence of prediabetes was the main outcome of this study, and we defined prediabetes using the HbA1c cut-off of 5.7–6.4%, identified by the American Diabetes Association [19]. We then separated our population into two groups: those with HbA1c levels more than and equal to or less than 5.7%; we defined participants who had HbA1c levels over 5.7% as those with prediabetes.

As the main independent variable, RHGS was calculated as the maximal absolute handgrip strength of both hands divided by BMI [11]. In the survey, handgrip strength was measured in the standing position in participants without disabilities in either hand. We considered sex-specific RHGS, because RHGS has been reported to differ according to sex [18]. We then divided sex-specific RHGS into quartiles and specified the 1st, 2nd, 3rd, and 4th quartiles as the low, mid-low, mid-high, and high groups, respectively.

Independent variables that may act as potential confounding variables included sociodemographic, economic, and health-related characteristics. Sociodemographic characteristics included age (19–29, 30–39, 40–49, 50–59, or ≥60 years), marital status (living with or without spouse), region (metropolitan or rural area), and educational level (middle school or less, high school, and college or over). Economic characteristics included occupation category and household income (low, mid-low, mid-high, or high). Health-related characteristics included smoking status, alcohol consumption status, aerobic exercise, BMI, waist circumference, and comorbidities.

Occupations were categorized according to the Korean version of the Standard Classification of Occupations (KSCO) based on the International Standard Classification of Occupations by the International Labor Organization. We re-categorized the classifications into four categories: white (office work), pink (sales and service), blue (agriculture, forestry, fishery, and armed forces occupation), and inoccupation. Lifetime smoking experience was classified as “yes” or “no” based on the response to the question: “Are you a current smoker?”. Drinking experience was classified as “yes” or “no” based on the response to the following question: “How often did you drink alcohol in the current year?”. Those who had consumed alcohol once or more within the current year were classified to have drinking experience. Those practicing aerobic exercise were defined as participants who performed moderate exercise for over 150 min, vigorous exercise for over 75 min, or a mixture of both (rating 1 min for moderate exercise and 2 min for vigorous exercise) for over 150 min per week regardless of working and exercising. BMI was classified into three groups as follows: underweight and normal range (≤22.9), overweight (23.0–24.9), and obese (≥25). Waist circumference (WC) was classified into two groups according to the cut-off point for central obesity for adults, recommended by the Korean Society for the Study of Obesity. The cut-off points are as follows: for men, ≥90 cm and for women, ≥85 cm. Comorbidities included hypertension, hyperlipidemia, stroke and myocardial infarction, and angina, and we calculated the number of comorbid diseases that each person had.

### Statistical analysis

Independent variables were compared using the chi-squared test to identify the association between RHGS and HbA1c levels. After adjusting for sociodemographic, economic, and health-related variables, we used multiple logistic regression analysis to evaluate the association between RHGS and HbA1c levels. Multiple logistic regression is used for dichotomous single-outcome variables and when there is more than one independent variable [20]. The results were reported using odds ratios (ORs) and confidence intervals (CIs). Moreover, we performed subgroup analysis stratified by sex and multiple logistic regression analysis to examine the associations of sex-specific RHGS in subjects with HbA1c levels, age, smoking status, alcohol consumption, aerobic exercise, BMI, and WC. In the subgroup analysis, we tested the trends for significance after adjusting for sociodemographic, economic, and health-related variables to determine the OR trend related to prediabetes (≥5.7%) in each category. Differences were considered significant at p-values of <0.05 as well as at p-values for trends <0.05. All statistical analyses were performed using SAS software (version 9.4; SAS Institute, Cary, NC).

## Results

For the purpose of this study, we analyzed each variable according to sex. Table 1 shows the general characteristics of the study population. Among the 13,384 participants, 1,643 men (28.2%) and 2,148 women (28.4%) met the criteria for prediabetes. According to RHGS, 572 (39.3%), 462 (31.8%), 367 (25.2%), and 242 (16.6%) men with prediabetes and 815 (43.1%), 584 (30.9%), 450 (23.8%), and 299 (15.8%) women with prediabetes were included in the low, mid-low, mid-high, and high RHGS groups, respectively.

**Table 1:** General characteristics of the study population.

Variables	HbA1c													
	Male							Female						
	TOTAL		≥5.7%		<5.7%		*P-value*	TOTAL		≥5.7%		<5.7%		*P-value*
	N	%	N	%	N	%		N	%	N	%	N	%	
**Total(n = 13,384)**	5,818	100.0	1,643	28.2	4,175	71.8		7,566	100.0	2,148	28.4	5,418	71.6	
**Sex-specific RHGS**^**a**^							<0.0001							<0.0001
Q1 (low)	1,455	25.0	572	39.3	883	60.7		1,892	25.0	815	43.1	1,077	56.9	
Q2 (mid-low)	1,454	25.0	462	31.8	992	68.2		1,891	25.0	584	30.9	1,307	69.1	
Q3 (mid-high)	1,455	25.0	367	25.2	1,088	74.8		1,891	25.0	450	23.8	1,441	76.2	
Q4 (high)	1,454	25.0	242	16.6	1,212	83.4		1,892	25.0	299	15.8	1,593	84.2	
**Age (years)**							<0.0001							<0.0001
19–29	902	15.5	45	5.0	857	95.0		1,024	13.5	38	3.7	986	96.3	
30–39	1,097	18.9	176	16.0	921	84.0		1,357	17.9	150	11.1	1,207	88.9	
40–49	1,140	19.6	287	25.2	853	74.8		1,592	21.0	296	18.6	1,296	81.4	
50–59	1,021	17.5	380	37.2	641	62.8		1,505	19.9	554	36.8	951	63.2	
≥60	1,658	28.5	755	45.5	903	54.5		2,088	27.6	1,110	53.2	978	46.8	
**Marital Status**							<0.0001							0.0047
Living with spouse	4,108	70.6	1,366	33.3	2,742	66.7		5,165	68.3	1,518	29.4	3,647	70.6	
Living without spouse	1,710	29.4	277	16.2	1,433	83.8		2,401	31.7	630	26.2	1,771	73.8	
**Region**							0.1971							0.9529
Metropolitan area	2,766	47.5	759	27.4	2,007	72.6		3,618	47.8	1,026	28.4	2,592	71.6	
Rural	3,052	52.5	884	29.0	2,168	71.0		3,948	52.2	1,122	28.4	2,826	71.6	
**Occupational categories**^**b**^							<0.0001							<0.0001
White	1,880	32.3	419	22.3	1,461	77.7		1,898	25.1	292	15.4	1,606	84.6	
Pink	610	10.5	151	24.8	459	75.2		1,174	15.5	349	29.7	825	70.3	
Blue	1,861	32.0	633	34.0	1,228	66.0		1,102	14.6	443	40.2	659	59.8	
Inoccupation	1,467	25.2	440	30.0	1,027	70.0		3,392	44.8	1,064	31.4	2,328	68.6	
**Educational level**							<0.0001							<0.0001
Middle school or less	1,172	20.1	509	43.4	663	56.6		2,159	28.5	1,035	47.9	1,124	52.1	
High school	2,016	34.7	531	26.3	1,485	73.7		2,438	32.2	610	25.0	1,828	75.0	
College or over	2,630	45.2	603	22.9	2,027	77.1		2,969	39.2	503	16.9	2,466	83.1	
**Household income**							<0.0001							<0.0001
Low	798	13.7	291	36.5	507	63.5		1,220	16.1	520	42.6	700	57.4	
Mid-low	1,346	23.1	422	31.4	924	68.6		1,826	24.1	560	30.7	1,266	69.3	
Mid-high	1,728	29.7	440	25.5	1,288	74.5		2,177	28.8	554	25.4	1,623	74.6	
High	1,946	33.4	490	25.2	1,456	74.8		2,343	31.0	514	21.9	1,829	78.1	
**Smoking**							<0.0001							<0.0001
Yes	4,339	74.6	1,354	31.2	2,985	68.8		859	11.4	167	19.4	692	80.6	
No	1,479	25.4	289	19.5	1,190	80.5		6,707	88.6	1,981	29.5	4,726	70.5	
**Alcohol consumption**							<0.0001							<0.0001
Yes	4,952	85.1	1,317	26.6	3,635	73.4		5,237	69.2	1,254	23.9	3,983	76.1	
No	866	14.9	326	37.6	540	62.4		2,329	30.8	894	38.4	1,435	61.6	
**Practicing aerobic exercise**							<0.0001							<0.0001
Yes	2,834	48.7	672	23.7	2,162	76.3		3,252	43.0	844	26.0	2,408	74.0	
No	2,984	51.3	971	32.5	2,013	67.5		4,314	57.0	1,304	30.2	3,010	69.8	
**Obesity Status (BMI)**^**c**^							<0.0001							<0.0001
Underweight & Normal range	1,989	34.2	396	19.9	1,593	80.1		4,012	53.0	786	19.6	3,226	80.4	
Overweight	1,532	26.3	416	27.2	1,116	72.8		1,548	20.5	511	33.0	1,037	67.0	
Obese	2,297	39.5	831	36.2	1,466	63.8		2,006	26.5	851	42.4	1,155	57.6	
**Waist circumference**^**d**^							<0.0001							<0.0001
Men(≥90cm), Women(≥85cm)	1,761	30.3	724	41.1	1,037	58.9		1,704	22.5	809	47.5	895	52.5	
Men(<90cm), Women(<85cm)	4,057	69.7	919	22.7	3,138	77.3		5,862	77.5	1,339	22.8	4,523	77.2	
**The number of chronic diseases**^**e**^							<0.0001							<0.0001
0	4,227	72.7	925	21.9	3,302	78.1		5,649	74.7	1,135	20.1	4,514	79.9	
1	1,076	18.5	469	43.6	607	56.4		1,283	17.0	623	48.6	660	51.4	
≥2	515	8.9	249	48.3	266	51.7		634	8.4	390	61.5	244	38.5	

^a^ Relative Hand Grip Strength, categorized into sex-specific quartiles

Men: Q1(<2.78), Q2(2.78–3.22), Q3(3.22–3.66), Q4(>3.66).

Women: Q1(<1.67), Q2(1.67–2.00), Q3(2.00–2.33), Q4(>2.33).

^b^Three groups (white, pink, blue) based on the International Standard Classification Occupations codes. Inoccupation group includes housewives.

^c^BMI: Body mass index/obesity status defined by BMI based on the 2018 Clinical Practice Guidelines for Overweight and Obesity in Korea.

^d^Central obesity is defined by waist circumference. The cut-off points for Korean adults were decided by the Korean Society for the Study of Obesity.

^e^Chronic disease was defined as diagnosed diseases: hypertension, hyperlipidemia, stroke and myocardial infarction or angina. The number of chronic diseases is the sum of the number of diagnosed above diseases.

Table 2 presents the factors associated with prediabetes. In men, the low and mid-low RHGS groups showed increased odds for prediabetes (low group, OR: 1.42, 95% CI: 1.10–1.82; mid-low group, OR: 1.32, 95% CI: 1.04–1.67). However, no female group showed a significant association between RHGS and prediabetes. Participants who were over the age of 30; qualified as overweight, obese, or having central obesity; and had comorbidities showed significant associations with prediabetes among both sexes.

**Table 2:** Factors associated with prediabetes.

Variables	HbA1c ≥5.7%							
	Male				Female			
	OR	95% CI			OR	95% CI		
**Sex-specific RHGS**^**a**^								
Q1 (low)	1.42	(1.10	-	1.82)	1.10	(0.86	-	1.40)
Q2 (mid-low)	1.32	(1.04	-	1.67)	0.95	(0.76	-	1.19)
Q3 (mid-high)	1.16	(0.91	-	1.48)	0.99	(0.81	-	1.21)
Q4 (high)	1.00				1.00			
**Age (years)**								
19–29	1.00				1.00			
30–39	3.36	(2.23	-	5.07)	2.85	(1.88	-	4.33)
40–49	5.85	(3.90	-	8.76)	5.63	(3.73	-	8.50)
50–59	9.46	(6.24	-	14.35)	12.93	(8.42	-	19.85)
≥60	11.47	(7.38	-	17.82)	20.17	(12.83	-	31.69)
**Marital status**								
Living with spouse	1.00				1.00			
Living without spouse	0.98	(0.79	-	1.20)	1.02	(0.86	-	1.21)
**Region**								
Metropolitan area	1.00				1.00			
Rural	0.87	(0.75	-	1.02)	0.87	(0.76	-	1.01)
**Occupational categories**^**b**^								
White	1.00				1.00			
Pink	1.33	(1.02	-	1.73)	1.13	(0.90	-	1.43)
Blue	1.29	(1.03	-	1.61)	1.17	(0.92	-	1.48)
Inoccupation	1.09	(0.85	-	1.39)	0.98	(0.81	-	1.18)
**Educational level**								
Middle school or less	1.03	(0.83	-	1.28)	1.30	(1.05	-	1.59)
High school	0.93	(0.73	-	1.19)	1.30	(1.01	-	1.66)
College or over	1.00				1.00			
**Household income**								
Low	1.10	(0.84	-	1.44)	1.14	(0.90	-	1.46)
Mid-low	1.09	(0.90	-	1.33)	1.11	(0.91	-	1.36)
Mid-high	0.92	(0.76	-	1.11)	1.13	(0.95	-	1.35)
High	1.00				1.00			
**Smoking**								
Yes	1.39	(1.16	-	1.66)	0.87	(0.70	-	1.09)
No	1.00				1.00			
**Alcohol consumption**								
Yes	0.80	(0.64	-	0.99)	0.85	(0.74	-	0.98)
No	1.00				1.00			
**Practicing aerobic exercise**								
Yes	1.00				1.00			
No	1.22	(1.05	-	1.42)	0.89	(0.77	-	1.02)
**Obesity status (BMI)**^**c**^								
Underweight and normal weight	1.00				1.00			
Overweight	1.30	(1.06	-	1.59)	1.33	(1.12	-	1.59)
Obese	1.86	(1.45	-	2.38)	1.55	(1.25	-	1.93)
**Waist circumference**^**d**^								
Men (≥90 cm), Women (≥85 cm)	1.66	(1.36	-	2.02)	1.62	(1.33	-	1.97)
Men (<90 cm), Women (<85 cm)	1.00				1.00			
**Number of chronic diseases**^**e**^								
0	1.00				1.00			
1	1.34	(1.12	-	1.61)	1.42	(1.20	-	1.67)
≥2	1.39	(1.08	-	1.78)	2.20	(1.77	-	2.74)

^a^ Relative hand grip strength, categorized into sex-specific quartiles

Men: Q1(<2.78), Q2(2.78–3.22), Q3(3.22–3.66), Q4(>3.66).

Women: Q1(<1.67), Q2(1.67–2.00), Q3(2.00–2.33), Q4(>2.33).

^b^Three groups (white, pink, blue) based on the International Standard Classification Occupations codes Inoccupation group includes housewives.

^c^BMI: Body mass index; obesity status was defined by BMI based on the 2018 Clinical Practice Guidelines for Overweight and Obesity in Korea.

^d^Central obesity is defined using waist circumference. The cut-off points for Korean adults were decided by the Korean Society for the Study of Obesity.

^e^Chronic diseases were defined as diagnosed diseases: hypertension, hyperlipidemia, stroke and myocardial infarction or angina. The number of chronic diseases is the sum of the number of diagnosed mentioned above.

Table 3 shows the results of subgroup analysis stratified by independent variables. Men who smoked, consumed alcohol, or had obesity in the low and mid-low RHGS groups showed significant positive associations with prediabetes. These results were consistent with the main results in Table 2. Moreover, men with central obesity who had lower RHGS showed higher associations with prediabetes (low group, OR: 2.40, 95% CI: 1.52–3.80; mid-low group, OR: 2.00, 95% CI: 1.26–3.17; mid-high group, OR: 1.76, 95% CI: 1.11–2.81), and a trend was observed (p = 0.0026). There were no significant differences in men, and no trend was seen.

**Table 3:** Subgroup analysis stratified by independent variables*.

Variables	HbA1c ≥5.7%													
	Sex-specific RHGS^**a**^													
	Q1 (low)				Q2 (mid-low)				Q3 (mid-high)				Q4 (high)	*P value* for trend
	OR	95% CI			OR	95% CI			OR	95% CI			OR	
**Male**														
**Age (years)**														
19–29	2.75	(0.70	-	10.83)	2.86	(0.90	-	9.11)	1.67	(0.42	-	6.57)	1.00	0.1143
30–39	2.06	(1.13	-	3.75)	1.79	(1.02	-	3.14)	1.43	(0.83	-	2.46)	1.00	0.0340
40–49	1.05	(0.60	-	1.82)	0.84	(0.53	-	1.34)	0.74	(0.46	-	1.19)	1.00	0.8438
50–59	1.62	(0.97	-	2.72)	1.81	(1.13	-	2.89)	1.51	(0.97	-	2.35)	1.00	0.0439
≥60	1.02	(0.65	-	1.63)	0.94	(0.59	-	1.50)	0.94	(0.56	-	1.57)	1.00	0.8427
**Smoking**														
Yes	1.35	(1.02	-	1.79)	1.31	(1.01	-	1.69)	1.12	(0.86	-	1.46)	1.00	0.1243
No	1.64	(0.91	-	2.94)	1.36	(0.77	-	2.40)	1.22	(0.71	-	2.10)	1.00	0.2343
**Alcohol consumption**														
Yes	1.50	(1.14	-	1.97)	1.43	(1.10	-	1.86)	1.22	(0.94	-	1.58)	1.00	0.0521
No	0.98	(0.52	-	1.83)	0.81	(0.44	-	1.47)	0.84	(0.45	-	1.57)	1.00	0.9467
**Practicing aerobic exercise**														
Yes	1.81	(1.23	-	2.67)	1.88	(1.32	-	2.68)	1.49	(1.02	-	2.16)	1.00	0.0078
No	1.17	(0.84	-	1.64)	1.00	(0.74	-	1.36)	0.96	(0.70	-	1.30)	1.00	0.7596
**Obesity status (BMI)**^**b**^														
Underweight and Normal range	1.06	(0.65	-	1.74)	0.92	(0.60	-	1.41)	1.10	(0.73	-	1.64)	1.00	0.6782
Overweight	0.91	(0.58	-	1.43)	1.11	(0.72	-	1.71)	1.07	(0.71	-	1.61)	1.00	0.4073
Obese	1.90	(1.23	-	2.92)	1.70	(1.12	-	2.60)	1.28	(0.82	-	1.99)	1.00	0.0034
**Waist circumference**^**c**^														
Men(≥90cm), Women(≥85cm)	2.40	(1.52	-	3.80)	2.00	(1.26	-	3.17)	1.76	(1.11	-	2.81)	1.00	0.0026
Men(<90cm), Women(<85cm)	1.01	(0.74	-	1.38)	1.14	(0.85	-	1.52)	1.03	(0.77	-	1.36)	1.00	0.4977
**Female**														
**Age (years)**														
19–29	0.63	(0.31	-	2.00)	1.38	(0.51	-	3.74)	0.20	(0.01	-	0.75)	1.00	0.9819
30–39	0.97	(0.45	-	2.06)	0.81	(0.42	-	1.56)	0.71	(0.41	-	1.23)	1.00	0.9816
40–49	1.42	(0.89	-	2.25)	0.76	(0.49	-	1.19)	0.83	(0.57	-	1.22)	1.00	0.3440
50–59	0.96	(0.62	-	1.48)	1.06	(0.72	-	1.55)	1.07	(0.75	-	1.53)	1.00	0.7479
≥60	1.09	(0.64	-	1.86)	0.89	(0.53	-	1.51)	1.21	(0.73	-	2.02)	1.00	0.9589
**Smoking**														
Yes	1.48	(0.72	-	3.03)	0.86	(0.42	-	1.74)	0.80	(0.44	-	1.47)	1.00	0.6348
No	1.10	(0.85	-	1.43)	0.97	(0.77	-	1.24)	1.01	(0.82	-	1.26)	1.00	0.7534
**Alcohol consumption**														
Yes	1.16	(0.86	-	1.58)	0.92	(0.70	-	1.21)	0.97	(0.76	-	1.23)	1.00	0.6670
No	1.03	(0.70	-	1.51)	0.99	(0.67	-	1.47)	0.99	(0.67	-	1.47)	1.00	0.9589
**Practicing aerobic exercise**														
Yes	1.17	(0.80	-	1.70)	0.87	(0.64	-	1.19)	0.97	(0.72	-	1.31)	1.00	0.7674
No	1.07	(0.77	-	1.49)	1.01	(0.74	-	1.39)	0.98	(0.74	-	1.29)	1.00	0.8685
**Obesity status (BMI)**^**b**^														
Underweight and Normal range	1.01	(0.72	-	1.41)	0.91	(0.67	-	1.23)	0.99	(0.78	-	1.26)	1.00	0.5765
Overweight	0.99	(0.60	-	1.63)	0.90	(0.56	-	1.44)	0.84	(0.52	-	1.35)	1.00	0.8835
Obese	1.03	(0.55	-	1.95)	0.82	(0.44	-	1.54)	0.84	(0.44	-	1.61)	1.00	0.3574
**Waist circumference**^**c**^														
Men(≥90cm), Women(≥85cm)	0.81	(0.44	-	1.51)	0.69	(0.37	-	1.30)	0.67	(0.36	-	1.27)	1.00	0.7709
Men(<90cm), Women(<85cm)	1.07	(0.81	-	1.41)	0.94	(0.73	-	1.21)	0.97	(0.78	-	1.21)	1.00	0.8557

*Adjusted for age, marital status, region, household income, job, educational status, smoking status, alcohol consumption, practicing aerobic exercise, BMI, waist circumference, and the number of chronic diseases.

^a^ Relative Hand Grip Strength, categorized into sex-specific quartiles

Men: Q1(<2.78), Q2(2.78–3.22), Q3(3.22–3.66), Q4(>3.66) / Women: Q1(<1.67), Q2(1.67–2.00), Q3(2.00–2.33), Q4(>2.33).

^b^BMI: Body mass index; obesity status was defined by BMI based on the 2018 Clinical Practice Guidelines for Overweight and Obesity in Korea.

^c^Central obesity is defined using waist circumference. The cut-off points for Korean adults were decided by the Korean Society for the Study of Obesity.

## Discussion

This study found that RHGS was negatively associated with prediabetes in men but not in women. This result is similar to that of a previous study, which found a negative relationship between handgrip strength and prediabetes in healthy-weight American adults without diabetes [21]. However, low and mid-low RHGS showed significant associations with prediabetes only in men in this study. This means that men with low handgrip strength relative to their BMI or with high BMI relative to their handgrip strength were likely to have an HbA1c level over 5.7%.

It is well-known that poor health behaviors lead to diabetes. For example, cigarette smoking is associated with increased insulin resistance [22], and excessive alcohol consumption increases the likelihood of developing of many diseases, including diabetes [23]. Moreover, type 2 diabetes is related to a decrease in physical activity and an increase in obesity [4]. Based on these findings, the significant association with prediabetes in men alone may be explained by health-related characteristics. The male participants included in this study had worse health behaviors than female participants. Men tended to smoke more, drink more alcohol, and have a higher prevalence of obesity or central obesity than women. This tendency was similar in the subgroup analysis in this study, which showed the relationship between RHGS, prediabetes, and health-related characteristics. We found that men who smoked, drank alcohol, or were obese had significant associations with lower RHGS and prediabetes. This might be caused by sex-specific demographic and sociographic characteristics: in South Korea, the proportion of men who smoke and drink is higher than that of women [24], and similar patterns have been seen in other countries [25, 26]. Our study’s results were consistent with those reported previously [27].

The differences in hormones and glucose metabolism could also explain the different results between the sexes. Leptin is affected by sex hormones, and high levels of leptin are related to an increased risk of diabetes in men but not in women [28]. Additionally, testosterone impairs insulin-mediated glucose uptake and leads to impaired glycogen synthase expression [29]. In terms of glucose metabolism, some studies have revealed that men had a higher prevalence of impaired fasting glucose levels, while women had a higher prevalence of impaired 2-hour plasma glucose levels [30]. This finding might have affected our results as our definition of diabetes did not consider impaired 2-hour plasma glucose levels. For these reasons, men were more likely to show a significant association between RHGS and prediabetes than women.

In the subgroup analysis, the trends of association were significant in men aged 30–39 years and 50–59 years, those who regularly performed aerobic exercises, those with obesity, and those with central obesity. Furthermore, the association between RHGS and prediabetes was stronger in men who had central obesity. A large WC is a well-known indicator for predicting non-insulin-dependent diabetes [31], and this finding shows that low muscle strength and central obesity are more relevant to diabetes than central obesity alone.

Another findings comparing mentioned above, among men, no drinking experience and lower RHGS were negatively associated with prediabetes, but this association was not significant. However, alcohol consumption is expected to have stronger association with prediabetes than low handgrip in this population. In contrast, not practicing aerobic exercise and having lower RHGS were positively associated with prediabetes among men, but not significantly. Therefore, it can be assumed that low handgrip is a stronger factor than performance of aerobic exercise in this population.

We suspected a negative relationship between RHGS and prediabetes based on the results of some studies which reported an association between muscle strength and diabetes. First, diabetes leads to muscle weakness due to insulin resistance and glucose toxicity. Moreover, insulin resistance is associated with impaired mitochondrial function in muscles [32], which is related to attenuated muscle strength. In addition, intermuscular adipose tissue volume is inversely related with physical function [33] and is greater in those with diabetes [34]. Based on these findings, muscle strengthening is needed to prevent diabetes.

This study has several strengths. By excluding those with diabetes or an HbA1c level over 6.5%, we determined the relationship between handgrip strength and prediabetes. Moreover, this provides evidence for easy prediction of diabetes by assessing both handgrip strength and BMI. In addition, we reported WC as a confounder in this study, which showed a more positive relationship between low muscle strength and prediabetes than with BMI. WC could also be used as a measure for exploring further risk factors of diabetes. Finally, we used representative data collected by a reliable institution in South Korea [35].

However, there are several limitations to this study. First, we used cross-sectional data, and thus, we could only determine an association between RHGS and prediabetes; we could not examine causality between these two variables. Further explanatory studies are needed to infer causality. Second, the estimation of handgrip strength can change according to measurement position [36]. Thus, before applying handgrip strength as a predictor of diabetes, a standardized and accurate method for measuring handgrip strength should be used for all participants. Third, there may be other residual factors that were not included in this study. Fourth, data on insulin resistance and hormone levels were not collected which could have provided a statistical explanation for the results. Fifth, the health-related characteristics used in this study, such as smoking and drinking status, were measured through self-reported questionnaires. Lastly, we used South Korean population-based data, and thus, our results may not apply to other ethnic groups.

## Conclusions

This study found a negative relationship between RHGS and prediabetes in men. Thus, an index combining handgrip strength and BMI would be a practical and inexpensive tool for predicting diabetes among men. In addition, central obesity showed a stronger relationship with low muscle strength and prediabetes than with BMI. Programs aimed at improving muscle strength or reducing WC would be beneficial as interventions for preventing diabetes. Further research such as longitudinal studies are required to investigate the causality of RHGS on prediabetes risk.
